# Targeting circulating labile heme as a defense strategy against malaria

**DOI:** 10.26508/lsa.202302276

**Published:** 2024-02-02

**Authors:** Susana Ramos, Viktoria Jeney, Ana Figueiredo, Tiago Paixão, Maria Rosário Sambo, Vatúsia Quinhentos, Rui Martins, Zélia Gouveia, Ana Rita Carlos, Ana Ferreira, Teresa F Pais, Hugo Lainé, Pedro Faísca, Sofia Rebelo, Silvia Cardoso, Emanuela Tolosano, Carlos Penha-Gonçalves, Miguel P Soares

**Affiliations:** 1 Instituto Gulbenkian de Ciência , Oeiras, Portugal; 2 Hospital Pediátrico David Bernardino, Luanda, Angola; 3 Faculdade de Medicina, Universidade Agostinho Neto, Luanda, Angola; 4 Department Molecular Biotechnology and Health Sciences, University of Torino, Torino, Italy

## Abstract

Severe presentations of malaria emerge as *Plasmodium (P.) spp.* parasites invade and lyse red blood cells (RBC), producing extracellular hemoglobin (HB), from which labile heme is released. Here, we tested whether scavenging of extracellular HB and/or labile heme, by haptoglobin (HP) and/or hemopexin (HPX), respectively, counter the pathogenesis of severe presentations of malaria. We found that circulating labile heme is an independent risk factor for cerebral and non-cerebral presentations of severe *P. falciparum* malaria in children. Labile heme was negatively correlated with circulating HP and HPX, which were, however, not risk factors for severe *P. falciparum* malaria. Genetic *Hp* and/or *Hpx* deletion in mice led to labile heme accumulation in plasma and kidneys, upon *Plasmodium infection*. This was associated with higher incidence of mortality and acute kidney injury (AKI) in ageing but not adult *Plasmodium*-infected mice, and was corroborated by an inverse correlation between heme and HPX with serological markers of AKI in *P. falciparum* malaria. In conclusion, HP and HPX act in an age-dependent manner to prevent the pathogenesis of severe presentation of malaria in mice and presumably in humans.

## Abstract (precis)

Malaria remains a major cause of human morbidity and mortality. Circulating labile heme is an independent risk factor for severe *P. falciparum* malaria, suggesting that labile heme may be a therapeutic target against severe malaria.

## Introduction

Malaria is an ancestral vector-borne disease, transmitted by the bite of female *Anopheles* mosquitoes. Upon injection into the dermis, *Plasmodium* sporozoites migrate to the liver, invading, developing and proliferating in hepatocytes (Prudencio et al, 2006). Clinical presentations occur later, during the blood stage of infection, as asexual stages of the parasite invade, develop, and proliferate in RBC.

The blood stage of *Plasmodium* infection is associated with a transient depletion of erythrophagocytic macrophages (Gazzinelli et al, 1988; Nahrendorf et al, 2021; Wu et al, 2023), which decouples RBC lysis from erythrophagocytosis and iron recycling (Wu et al, 2023). The ensuing intravascular hemolysis releases HB α_2_β_2_ tetramers, which disassemble into αβ dimers in plasma, releasing their non-covalently bound prosthetic heme groups (Pamplona et al, 2007; Gouveia et al, 2017). Labile heme refers to the pool of circulating heme, loosely bound to plasma proteins and macromolecules (Gouveia et al, 2017; Ramos et al, 2019), which fails to control the redox activity of the iron contained in the protoporphyrin ring.

Labile heme is an alarmin (Ferreira et al, 2008; Soares & Bozza, 2016) that catalyzes the pathogenesis of severe and often lethal outcomes of experimental malaria in mice (Pamplona et al, 2007; Ferreira et al, 2008; Gozzelino et al, 2010; Ramos et al, 2019; Wu et al, 2023). The pathogenic effects of labile heme are countered by the infected host, via the induction of heme oxygenase-1 (HO-1) (Pamplona et al, 2007; Ferreira et al, 2008; Gozzelino et al, 2010; Ramos et al, 2019), a stress responsive enzyme that cleaves the protoporphyrin ring of heme and generates equimolar amounts of biliverdin, iron, and carbon monoxide (Tenhunen et al, 1968; Gozzelino et al, 2010). This defense strategy was co-opted throughout evolution to prevent the onset of severe presentations of malaria in individuals carrying a single (i.e. hemizygous)-sickle HB mutation (i.e. sickle trait) (Ferreira et al, 2011).

Mammals express a number of plasma proteins that limit the release of heme from extracellular HB or restrain the redox activity of labile heme (Ferreira et al, 2008; De Simone et al, 2023). These include the HB scavenger haptoglobin (HP) and the heme scavengers, hemopexin (HPX), and ⍺1-microglobulin (De Simone et al, 2023), respectively. The observation that HP and HPX limit the extent of renal damage imposed by sterile intravascular hemolysis (Tolosano et al, 1999), suggested that HP and HPX might counter the pathogenesis of malaria-associated AKI, a major independent risk factor for *P. falciparum* malaria mortality, in children and adults (Sitprija, 1988; Trang et al, 1992; Mishra & Das, 2008; Plewes et al, 2017; Cruz et al, 2018; Katsoulis et al, 2021; Wu et al, 2023).

The HP protein complex is composed of two αβ dimers (∼100–160 kD), generated by proteolytic cleavage of a common precursor, linked by disulphide bridges (Polticelli et al, 2008; Andersen et al, 2017). Humans carry two allelic *HP1* and *HP2* variants, expressing three *HP1-1*, *HP2-1*, and *HP2-2* genotypes, with affinities towards αβ HB dimers, in the range of K_M_∼10^−12^ M. Binding of HP to αβ HB (Andersen et al, 2012), restrains HB oxidation and the release of its prosthetic heme groups (Andersen et al, 2012, 2017; Schaer et al, 2013). HB/HP complexes are scavenged, via CD163, by erythrophagocytic macrophages, coupling HB disposal with heme catabolism by HO-1.

*P. falciparum* malaria has been associated with depletion of circulating HP in children (Trape et al, 1985), presumably because of the removal of HP/HB complexes by macrophages. Whether or not the allelic *HP1* and *HP2* variants are associated with *P. falciparum* malaria incidence and/or outcome is not clear. The *HP1-1* genotype, with the highest HB affinity, was linked with *P. falciparum* malaria susceptibility and severe disease (Quaye et al, 2000), whereas other studies suggest the *HP1-2* (Elagib et al, 1998) and *HP2-2* (Atkinson et al, 2007) genotypes, with an intermediate and lowest HB affinity, respectively, are correlated with higher risk of severe *P. falciparum* malaria. Of note, genetic deletion of *Hp* in mice was associated with increased parasite burden (Hunt et al, 2001).

HPX is a 63-kD plasma protein that binds labile heme with the highest affinity (K_d_ < 10^−12^ M) of any protein described so far (Muller-Eberhard, 1970; Paoli et al, 1999) and neutralizes heme cytotoxicity (Larsen et al, 2010). Heme/HPX complexes are removed from the circulation via the low-density lipoprotein receptor-related protein 1 (LRP-1/CD91), expressed by circulating monocytes. At least one study suggests that the ratio of plasma heme to HPX is associated with *P. falciparum* malaria severity in children (Elphinstone et al, 2016). Other studies have also reported an association between plasma heme and extracellular HB with severe *P. falciparum* in children (Elphinstone et al, 2015) and *P. vivax* in adults (Mendonca et al, 2012).

To determine whether the HP/HB and/or HPX/heme scavenging systems are protective against malaria we combined the analyzes of a pediatric *P. falciparum* malaria case-control study (Sambo et al, 2010) with experimental models of malaria in mice carrying *Hp* and/or *Hpx* genetic deletions. We found that labile heme is an independent risk factor for cerebral and non-cerebral presentations of severe *P. falciparum* malaria and that HP and HPX act in an age-depended manner to prevent the pathogenesis of non-cerebral severe malaria in mice. Neither HP, HPX nor labile heme interfere with parasite burden, suggesting that the HP/HB and HPX/heme scavenging systems contribute to the establishment of disease tolerance to malaria (Medzhitov et al, 2012; Martins et al, 2019). These findings suggest that HP and/or HPX genetic variants may contribute to age-dependent increase in malaria susceptibility (Dondorp et al, 2008).

## Results

### Labile heme is an independent risk factor of severe *P. falciparum* malaria

**Figure 1.**
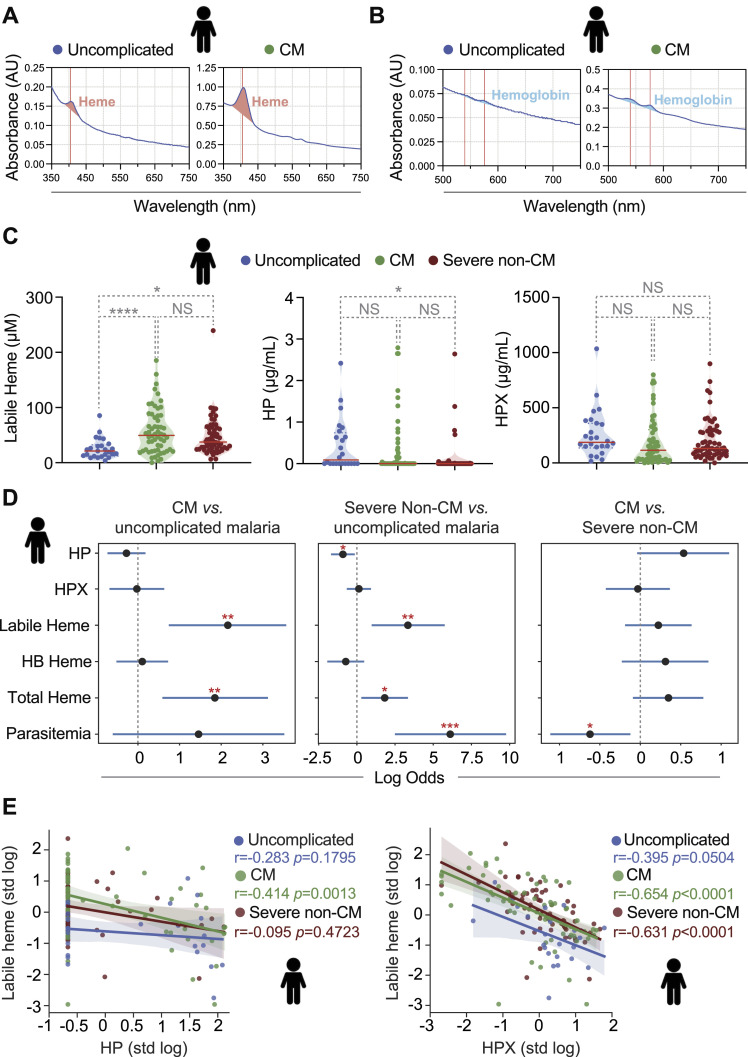
Heme accumulation in serum correlates with *P. falciparum* malaria severity. **(A, B)** Representative UV–visible spectra of plasma from *P. falciparum*-infected individuals with uncomplicated malaria or developing CM, highlighting (A) Soret region (λ_363–383_ nm) corresponding to labile heme, with a peak at λ_405_ nm and (B) λ_582_ nm region corresponding to HB-bound heme. **(C)** Labile heme, haptoglobin (HP), hemopexin (HPX) concentrations in serum from *P. falciparum*-infected individuals stratified according to disease severity: Uncomplicated malaria (N = 25), cerebral malaria (CM; N = 58) or severe non-cerebral malaria (N = 61). Circles represent individuals and red lines indicate median values. *P*-values determined using a one-way ANOVA test and subsequent posthoc tests. NS, nonsignificant; *P < 0.05; ****P < 0.0001. **(C, D)** Contribution of each of the parameters to distinguish the indicated sub-groups (stratified as in (C)), controlling for age and sex. Values indicate regression coefficients of the standardized variable on a logit regression. Raw data in Table 1. **(E)** Correlation coefficients between log-transformed haptoglobin (HP; left panel) or hemopexin (HPX; right panel) versus labile heme concentration in serum of *P. falciparum*-infected individuals, stratified as in (C). Circles represent individual patients. *P*-values determined using a Spearman’s rank correlation coefficient test. Spearman’s correlation coefficients (r) are highlighted. Source data are available for this figure.

We analyzed a case-control study of *P. falciparum*-infected children, ranging from 6 mo old to 13 yr old, hospitalized at Hospital Pediátrico David Bernardino, Luanda, Angola (Sambo et al, 2010). The original study included 130 cases of cerebral malaria (CM), 158 cases of severe non-cerebral, 142 cases of uncomplicated malaria and 319 children not infected by *P. falciparum*, selected randomly from the vaccination ward (Sambo et al, 2010). A subgroup of 58 cases of CM, 61 cases of severe non-cerebral malaria and 25 uncomplicated cases of malaria, for which serum was available, was evaluated for HP, HPX, total heme (i.e., HB-bound heme plus heme bound to other serum proteins and macromolecules), HB-bound heme and labile heme (i.e., fraction of total heme bound to serum proteins and macromolecules other than HB; Total heme - HB-bound heme) concentration in serum (Table 1; Fig 1A and B).

**Table 1.** Baseline demographic and serological characteristics of *P. falciparum*-infected children according to disease severity.

	Total	Uncomplicated	CM	Severe non-cerebral	*P*-value	Test
	**n = 144**	**n = 25**	**n = 58**	**n = 61**		
Sex (M/F)	68/56	5/9	35/22	28/25	*P* = 0.21	chi2
Age (months)	43.0 (22.5–55.5) n = 123	51.0 (18.75–78.5) n = 14	43.0 (24.0–70.5) n = 56	36.0 (22.0–49.0) n = 53	*P* = 0.24	kruskal-wallis
Parasitaemia	125.0 (14.0–298.0) n = 123	11.5 (3.25–38.25) n = 14	27.5 (4.5–200.0) n = 56	210.0 (150.0–380.0) n = 53	***P* < 0.001** ^b^ ^,^ ^c^	kruskal-wallis
Total Heme (μM)	53.7 (33.59–76.95) n = 143	40.2 (22.8–50.5) n = 25	66.2 (43.32–100.18) n = 58	51.1 (34.85–76.65) n = 60	***P* < 0.001** ^a^ ^,^ ^b^	kruskal-wallis
Hb Heme (μM)	9.8 (5.2–18.4) n = 142	10.8 (7.26–18.8) n = 25	8.9 (5.54–19.73) n = 58	10.3 (5.11–15.75) n = 59	*P* = 0.69	kruskal-wallis
Labile Heme (μM)	38.1 (21.91–63.83) n = 143	21.4 (12.8–30.54) n = 25	49.5 (21.96–84.29) n = 58	37.5 (25.31–61.8) n = 60	***P* < 0.001** ^a^ ^,^ ^b^	kruskal-wallis
Hemopexin (μg/ml)	150.7 (63.11–297.02) n = 143	199.1 (150.73–359.9) n = 25	116.1 (33.81–293.21) n = 58	132.4 (84.66–289.84) n = 60	*P* = 0.0513	kruskal-wallis
Haptoglobin (μg/ml)	0.0 (0.0–0.05) n = 143	0.1 (0.0–0.67) n = 24	0.0 (0.0–0.16) n = 58	0.0 (0.0–0.0) n = 61	***P* = 0.003** ^b^	kruskal-wallis
LCN2 (ng/ml)	130.9 (87.82–195.15) n = 138	106.0 (50.04–161.2) n = 23	155.3 (93.93–258.63) n = 56	127.2 (98.08–165.93) n = 59	*P* = 0.05599	kruskal-wallis
Creatinine (mg/dl)	0.6 (0.46–0.9) n = 105	1.0 (0.85–1.19) n = 10	0.7 (0.47–0.92) n = 56	0.5 (0.44–0.72) n = 39	***P* = 0.0032** ^b^	kruskal-wallis

Except for sex, all values are median and interquartile range (IQR); CM, Cerebral Malaria.

a Statistical significance comparing uncomplicated versus CM.

b Statistical significance comparing uncomplicated versus severe non-cerebral.

c Statistical significance comparing CM versus severe non-cerebral.

CM and severe non-cerebral malaria were associated with a median concentration of labile heme in serum of 49.5 and 37.5 μM, respectively (Table 1; Fig 1C). This was significantly higher compared with the 21.4 μM median concentration of circulating labile heme in uncomplicated *P. falciparum* malaria (Table 1; Fig 1C). The median concentration of circulating labile heme was indistinguishable in CM versus severe non-cerebral malaria (Table 1; Fig 1C). These observations suggest that the accumulation of circulating labile heme is associated with the onset of CM and severe non-cerebral *P. falciparum* malaria, similar to experimental models of CM (Pamplona et al, 2007) and non-cerebral malaria (Seixas et al, 2009; Gouveia et al, 2017; Ramos et al, 2019) in mice.

The concentrations of labile and total heme in serum were major independent risk factors for *P. falciparum* CM versus uncomplicated malaria, when controlling for age and sex (Table 1, Fig 1D). This was also the case when comparing severe non-cerebral versus uncomplicated malaria (Table 1, Fig 1D). Moreover, labile heme remained significantly associated with *P. falciparum* CM versus uncomplicated malaria, even when controlling for parasitemia (*P* = 0.004).

Parasitemia was not a risk factor for CM versus uncomplicated *P. falciparum* malaria (Table 1, Fig 1D), similar to experimental rodent models of CM (Pamplona et al, 2007; Ferreira et al, 2008, 2011; Jeney et al, 2014). This suggests that the pathogenesis of *P. falciparum* CM is fueled, irrespectively of parasite burden, by the accumulation of circulating labile heme.

Parasitemia was an independent risk factor of severe *P. falciparum* non-cerebral versus uncomplicated malaria (Table 1, Fig 1D). This is consistent with the pathogenesis of life-threatening malaria anemia being fueled by the accumulation of high levels of labile heme in plasma owed to high parasite burdens and hemolysis (Seixas et al, 2009; Ramos et al, 2019; Ramos et al, 2022; Wu et al, 2023). Parasitemia was also an independent risk factor for severe non-cerebral malaria versus CM (Table 1, Fig 1D).

### HP and HPX are not risk factors of severe *P. falciparum* malaria

The concentrations of HP and HPX in serum were indistinguishable in children that developed *P. falciparum* CM versus those with uncomplicated malaria (Table 1, Fig 1C). In contrast, severe non-cerebral malaria was associated with lower concentration of circulating HP, but not HPX, compared with uncomplicated malaria (Table 1, Fig 1C). This is consistent with severe non-cerebral malaria being related with extensive hemolysis and accumulation of extracellular HB, presumably leading to HP depletion. Circulating HP was associated (*P* = 0.005) with the distinction between severe non-cerebral, but not CM, and uncomplicated malaria, when controlling for age and sex (Table 1, Fig 1D). HPX was not a raw risk factor for CM or severe non-cerebral malaria (Table 1, Fig 1D).

### HP and HPX are negatively correlated with labile heme in *P. falciparum* malaria

We asked whether HP and HPX are linked to the accumulation of labile heme in serum during *P. falciparum* malaria. In support of this hypothesis, children that developed CM showed a negative correlation between circulating HP and labile heme (Fig 1E). This was not observed in children that developed severe non-cerebral malaria or in uncomplicated malaria (Fig 1E). The negative correlation between HP and labile heme in children that developed CM remained significant after controlling for parasitemia (*P* < 0.013). These observations are consistent with HP regulating the levels of circulating labile heme in children that develop CM, irrespectively of parasite burden.

HPX was negatively correlated with circulating labile heme concentration in children that developed CM and severe non-cerebral malaria, but not in those with uncomplicated *P. falciparum* malaria (Fig 1E). The negative correlation between HPX and labile heme in CM (*P* < 0.0001) or in severe non-cerebral malaria (*P* < 0.0001) remained significant after controlling for parasitemia. This is consistent with HPX regulating the levels of labile heme in CM and severe non-cerebral patients, irrespectively of parasite burden.

Taken together, these observations are consistent with (1) hemolysis associated with *P. falciparum* malaria depleting circulating HP and HPX and (2) HP and HPX exerting some level of control over the accumulation of labile heme in serum, without preventing the onset of severe presentations of *P. falciparum* malaria.

### HP and HPX control the accumulation of labile heme in experimental rodent malaria

The protective effect exerted by HP and HPX against sterile intravascular hemolysis in mice (Tolosano et al, 2002) led us to hypothesize that HP and HPX may be protective against malaria-associated intravascular hemolysis. We tested this hypothesis in 8–12 wk-old (i.e., adult) C57BL/6 mice infected with *Plasmodium chabaudi chabaudi* AS (*Pcc*), a non-lethal experimental model of severe non-cerebral malaria associated with high parasite burdens (Seixas et al, 2009; Jeney et al, 2014), intravascular hemolysis (Gouveia et al, 2017; Ramos et al, 2019) and severe anemia (Wu et al, 2023).

**Figure 2.**
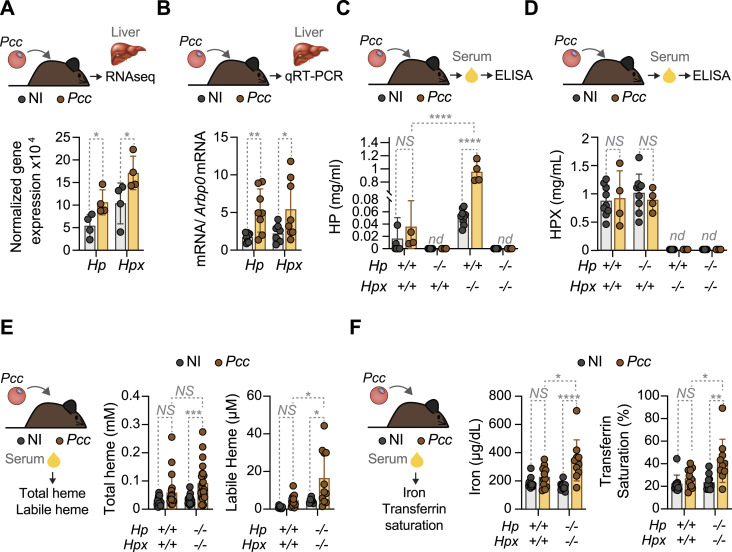
HP and HPX regulate serum labile heme accumulation during malaria. Mice were infected with *Pcc* (2 × 10^6^ iRBC) and serum was collected at the peak of parasitemia (day 6–7 post infection). **(A, B)** Relative expression of hepatic haptoglobin (*Hp*) and hemopexin (*Hpx*) mRNA in C57BL/6 mice, not infected (NI) or infected with *Pcc*, determined by (A) bulk RNAseq (N = 4 *per* genotype) from a previously published dataset (Ramos et al, 2022) or by (B) qRT–PCR from whole liver, normalized to *Arbp0* mRNA (N = 7 *per* genotype). **(C, D, E, F)** Serum concentrations of (C) haptoglobin (HP) and (D) hemopexin (HPX), (E) total heme (Left panel) and labile heme (Right panel), (F) Iron (Left panel) and transferrin saturation (Right panel), in *Pcc*-infected (1 × 10^5^ iRBC, at the peak of parasitemia: days 8–9 post-infection) and non-infected control mice (N = 10–12 *per* indicated genotype). Data represented as mean ± SD from one or two independent experiments with similar trend. Dots correspond to individual mice. *P*-values determined by two-way ANOVA. NS, non-significant; *P < 0.05; **P < 0.01; ***P < 0.001; ****P < 0.0001. Source data are available for this figure.

*Pcc* infection was associated with a relative increase in hepatic *Hp* and *Hpx* mRNA expression, compared with non-infected control mice, as assessed in a previously published RNA-seq data set (Fig 2A) (Ramos et al, 2022) and confirmed independently by qRT–PCR (Fig 2B). This was not linked however, with a corresponding increase in circulating HP and HPX levels (Fig 2C and D), presumably reflecting the “removal” of circulating HP/HB and HPX/heme complexes generated via intravascular hemolysis.

To address whether HP and/or HPX control the levels of circulating labile heme during *Plasmodium* infection we generated C57BL/6 mice carrying individual or combined germline *Hp* (*Hp*^−/−^), *Hpx* (*Hpx*^−/−^) or *Hp* and *Hpx* (*Hp*^−/−^*Hpx*^−/−^) gene deletions. These were confirmed by the quantification of circulating HP (Fig 2C) and/or HPX (Fig 2D) protein in serum.

*Hpx* deletion was associated with an increase in HP concentration in serum, as compared with age-matched control *Hp*^*+/+*^*Hpx*^*+/+*^ mice, both at steady state and after *Pcc* infection (Fig 2C). In contrast, *Hp* deletion had no impact on HPX concentration in serum, compared with age-matched control *Hp*^*+/+*^*Hpx*^*+/+*^ mice (Fig 2D). This suggests the existence of a crosstalk in the regulation of HP and HPX expression, whereby a reduction of HPX induces the expression of HP.

Labile heme concentration in serum was higher in *Pcc*-infected *Hp*^−/−^*Hpx*^−/−^ versus *Hp*^*+/+*^*Hpx*^*+/+*^ mice (Fig 2E). The concentration of total and labile heme in serum was similar at steady state in adult *Hp*^−/−^*Hpx*^−/−^ versus control *Hp*^*+/+*^*Hpx*^*+/+*^ mice (Fig 2E). This suggests that in adult mice the accumulation of labile heme in serum during malaria is controlled by HP and HPX.

We noticed a relatively lower accumulation of labile heme in serum during *Pcc* infection, compared with our previous studies (Gouveia et al, 2017; Ramos et al, 2019). This is likely attributed to the lower *Pcc* inoculum used in the present study.

Iron concentration in serum was higher in adult *Hp*^−/−^*Hpx*^−/−^ versus *Hp*^*+/+*^*Hpx*^*+/+*^ mice, infected by *Pcc*, but not at steady state (Fig 2F). Transferrin saturation, a pathophysiologic parameter reporting on circulating iron transport, was also higher in adult *Pcc*-infected *Hp*^−/−^*Hpx*^−/−^ versus *Hp*^*+/+*^*Hpx*^*+/+*^ mice (Fig 2F), but not at steady state. This suggests that HP and HPX control systemic iron metabolism during malaria in adult mice.

### HP and HPX are not essential to survive malaria in adult mice

**Figure 3.**
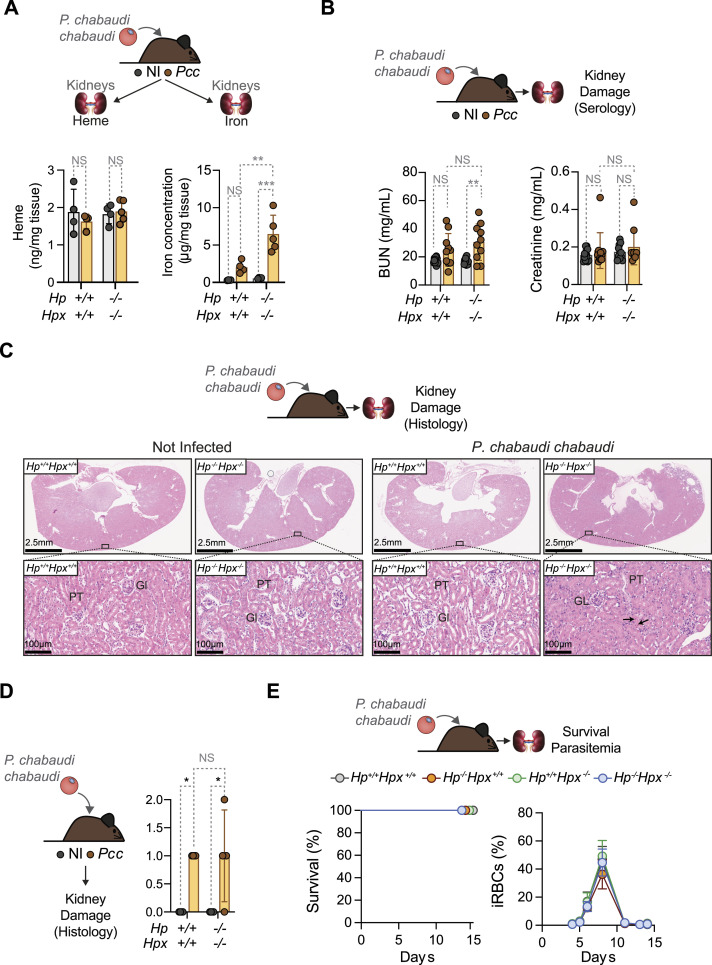
HP and HPX are not required to prevent malaria acute kidney injury in adult mice. Adult 8–12 wk-old mice from the indicated genotypes were infected with *Pcc* and serum was collected, at the peak of parasitemia. **(A)** Renal heme (Left panel) and iron (Right panel) concentrations. Mice were infected with 2 × 10^6^ iRBC and collected 7 d post infection. Data represented as mean ± SD from two independent experiments with a similar trend (N = 4–5 *per* genotype). Dots correspond to individual mice. **(B)** Blood urea nitrogen (left panel) and creatinine (right panel) concentrations in serum. Mice were infected with 1 × 10^5^ iRBC and collected 8–9 d post infection. Data represented as mean ± SD from two independent experiments. with similar trend (N = 7–12 *per* genotype). Dots correspond to individual mice. **(C)** Kidney H&E staining, representative of N = 4 mice *per* genotype, corresponding to *Pcc*-infected (1 × 10^5^ iRBC, at the peak of parasitemia: days 8–9 post-infection) and noninfected control mice. Top panels show whole kidney sections and bottom panels higher magnifications of the highlighted area. Arrowheads indicate renal proximal tubule epithelial single cell necrosis. GL, glomerulus; PT, proximal tubules. **(C, D)** Histopathological evaluation of the kidneys from (C) performed using digitalized whole-slide images, corresponding to whole kidney sections. Scores are represented as mean ± SD (n = 4–5 mice *per* genotype). Dots correspond to individual mice. Scores: 0 = No lesions; 1 = Single cell necrosis, discrete hemoglobin tubular casts; 2 = Mild; 3 = Moderate; 4 = Severe tubular cell necrosis, hemoglobin tubular casts. **(E)** Survival (left panel) and parasitemia (right panel) of *Pcc*-infected (1 × 10^5^ iRBC) *Hp*^*+/+*^*Hpx*^*+/+*^, *Hp*^*−/−*^*Hpx*^*+/+*^, *Hp*^*+/+*^*Hpx*^*−/−*^ and *Hp*^*−/−*^*Hpx*^*−/−*^ mice (N = 7–11 mice per genotype), from two independent experiments with similar trend. Survival is represented in Kaplan–Meier plots and parasitemia as mean ± SD. *P*-values in (A, B, D) determined by two-way ANOVA. NS, non-significant; *P < 0.05; **P < 0.01; ***P < 0.001; ****P < 0.0001. Source data are available for this figure.

Next, we asked whether HP and/or HPX counter the development of malaria-associated AKI. Renal heme content was similar in adult *Hp*^*−/−*^*Hpx*^*−/−*^ versus *Hp*^*+/+*^*Hpx*^*+/+*^ mice, both at steady state and after *Pcc* infection (Fig 3A). In contrast, renal iron overload was exacerbated in adult *Hp*^*−/−*^*Hpx*^*−/−*^ versus age-matched control *Hp*^*+/+*^*Hpx*^*+/+*^ mice infected with *Pcc* but not at steady state (Fig 3A). Renal iron overload was not associated however, with the development of AKI, as assessed by blood urea nitrogen and creatinine concentration in serum at the peak of *Pcc* infection (Fig 3B). This was confirmed histologically by the extent and frequency of HB casts and proximal tubular necrosis (Fig 3C and D).

**Figure 4.**
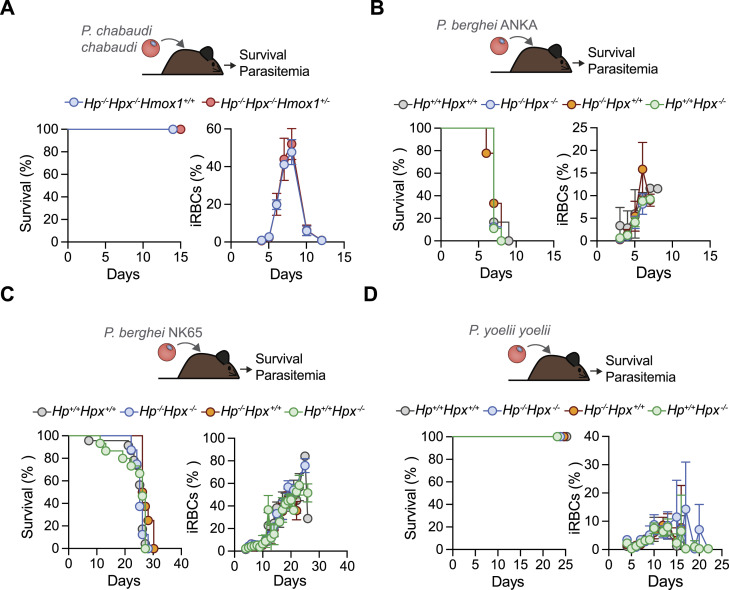
HP and HPX are not required to prevent malaria mortality in adult mice. Adult 8–12 wk-old mice from the genotypes indicated were infected with different *Plasmodium* strains (i.p., 1 × 10^5^ iRBC). **(A, B, C, D)** Survival (left panels) and parasitemia (right panels) were monitored daily from day 3 post infection with: (A) *P. chabaudi chabaudi* (*Pcc*; N = 8–10 mice, two independent experiments), (B) *P. berghei* ANKA-GFP (*Pb*ANKA; N = 6–9 mice, two independent experiments), (C) *P. berghei* NK65 (*Pb*NK65; N = 8–23 mice, five independent experiments), (D) *P. yoelii yoelii* 17XNL (*Pyy*17XN*L*; N = 8–11 mice, two independent experiments). Survival is represented in Kaplan–Meier plots. Percentage of infected RBCs (iRBC) was quantified by FACS when using *PbANKA*-GFP transgenic parasites, or by morphologic assessment of Giemsa-stained blood smears (four to five fields) when using other *Plasmodium* strains and is represented as mean ± SD. Source data are available for this figure.

Adult *Pcc*-infected *Hp*^*−/−*^*Hpx*^*−/−*^ mice survived and cleared parasitemia, similar to age-matched control *Pcc*-infected *Hp*^*+/+*^*Hpx*^*+/+*^ mice (Fig 3E). This was also the case for *Hp*^*−/−*^*Hpx*^*−/−*^*Hmox1*^*+/−*^ mice, lacking one *Hmox1* allele (Fig 4A), suggesting that the extent of renal iron overload imposed by HP and HPX depletion is not sufficient to precipitate the pathogenesis of malaria AKI in adult mice.

The virulence of other rodent-infective *Plasmodium* strains was also similar in adult *Hp*^*−/−*^*Hpx*^*−/−*^ versus age-matched control *Hp*^*+/+*^*Hpx*^*+/+*^ mice, as assessed for *P. berghei ANKA* (Fig 4B) or *P. berghei NK65* infection, with the latter failing to elicit experimental CM in *Hp*^*−/−*^*Hpx*^*−/−*^ mice (Fig 4C). Similar findings were obtained for *P. yoelii yoelii* infection, which was not lethal to adult *Hp*^*−/−*^*Hpx*^*−/−*^ mice nor to age-matched control *Hp*^*+/+*^*Hpx*^*+/+*^ mice (Fig 4D). This suggests that, in contrast to other components of the heme/iron detoxifying pathway, including HO-1 (Pamplona et al, 2007; Ferreira et al, 2008; Seixas et al, 2009; Jeney et al, 2014; Ramos et al, 2019, 2022; Wu et al, 2023), ferritin H chain (Gozzelino et al, 2012; Ramos et al, 2019) or ferroportin 1 (Zhang et al, 2018; Wu et al, 2023), HP and HPX are not essential to survive malaria in adult mice.

### Compensatory heme scavenging mechanisms during malaria

**Figure 5.**
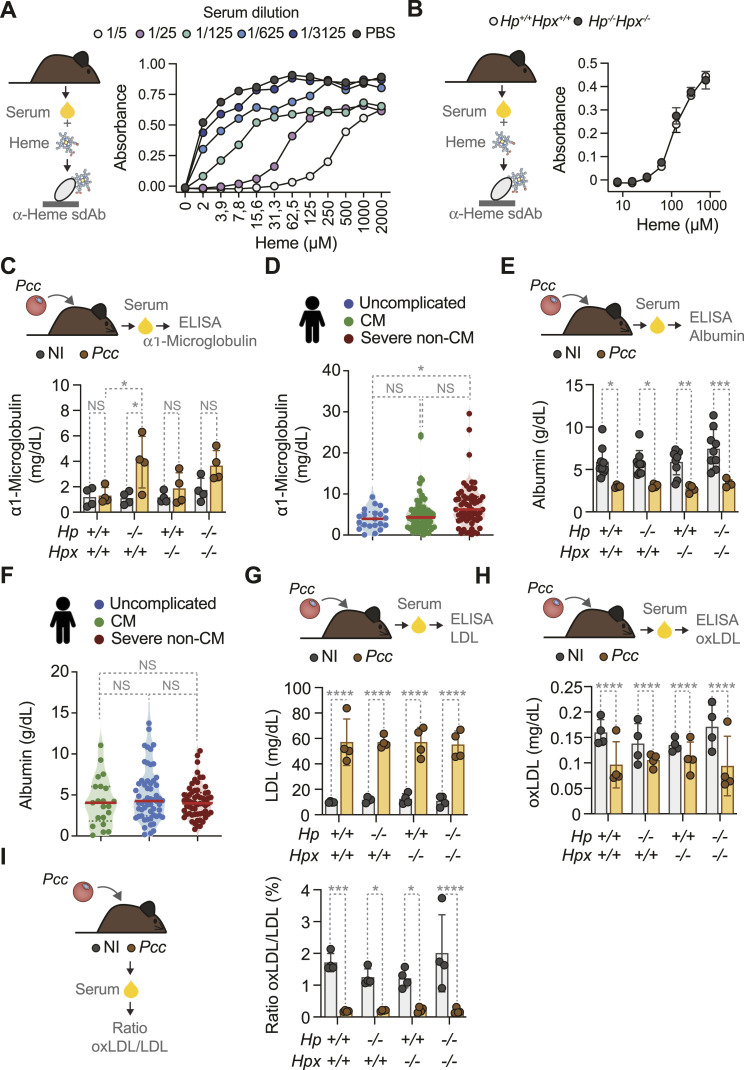
Compensatory heme scavenging mechanisms during malaria. **(A)** Heme buffering capacity of serum from C57BL/6 mice at steady state, assayed by a heme-specific single domain Ab-based sandwich ELISA. Hemin, at the concentrations indicated, was preincubated with serially diluted serum from C57BL/6 mice. Each dot represents a single well in one experiment. **(B)** Comparison of the heme buffering capacity of serum from *Hp*^*+/+*^*Hpx*^*+/+*^ versus *Hp*^*−/−*^*Hpx*^*−/−*^ mice, at steady state. **(A)** Increasing heme concentrations were pre-incubated with serum (1/250) in the same assay as in (A). Data shown as mean ± SD (N = 3 *per* genotype) from one experiment. **(C)** Serum concentrations of ⍺1-microglobulin (mg/dl) in *Pcc*-infected (1 × 10^5^ iRBC, at the peak of parasitemia: days 8–9 post-infection) and non-infected control mice (N = 4 *per* indicated genotype). **(D)** α1-Microglobulin concentrations in serum from *P. falciparum*-infected individuals stratified according to disease severity: Uncomplicated malaria (N = 21), cerebral malaria (CM; N = 58) or severe non-cerebral malaria (N = 60). **(C, E)** Serum concentrations of albumin in the same mice as in (C). **(D, F)** α1-Microglobulin concentrations in serum from *P. falciparum*-infected individuals as in (D). **(G, H, I)** Serum concentrations of (G) low density lipoprotein (LDL); (H) Oxidized LDL. **(G, H, I)** Ratio of oxidized LDL/LDL calculated from (G) and (H). Data in (C, E, G, H, I) represented as mean ± SD (N = 4 mice *per* genotype) from one to two independent experiments with similar trend. Dots represent individual mice. Data in (D, F) are represented in violin plots where circles represent individuals and red line indicates median values. **(D, F)** In (C, E, G, H, I) *P*-values were determined by two-way ANOVA and in (D, F) by one-way ANOVA. NS, nonsignificant; *P < 0.05; **P < 0.01; ***P < 0.001; ****P < 0.0001. Source data are available for this figure.

We then asked whether other plasma proteins and/or macromolecules might scavenge labile heme in the absence of HP and/or HPX. To this aim, we used an ELISA-based assay that quantifies serum heme buffering capacity, that is, the relative capacity of serum proteins and macromolecules to scavenge labile heme (Gouveia et al, 2017). The assay is based on a heme-specific single-domain antibody (sdAb) that binds heme with an affinity of 10^−7^ M (Gouveia et al, 2017). Heme binding to this sdAb was inhibited when heme was preincubated with serum from control *Hp*^*+/+*^*Hpx*^*+/+*^ mice (Fig 5A). This effect was dose dependent, that is, the higher the serum dilution and/or the amount of heme, the higher was heme recognition by the sdAb (Fig 5A). Surprisingly, the heme buffering capacity of serum from adult *Hp*^*−/−*^*Hpx*^*−/−*^ mice was indistinguishable from that of control *Hp*^*+/+*^*Hpx*^*+/+*^ mice, at steady state (Fig 5B). This suggests that circulating proteins and/or macromolecules other than HP and/or HPX can scavenge labile heme.

We next quantified other known serum heme-binding proteins and macromolecules, at the peak of *Pcc* infection. The concentration of the heme scavenger ⍺1-microglobulin (Allhorn et al, 2002) in serum from *Pcc*-infected mice was similar to that of control noninfected mice (Fig 5C). *Pcc*-infected *Hp*^*−/−*^ mice had higher concentrations of ⍺1-microglobulin in serum, compared with noninfected genotype-matched controls (Fig 5C). This suggests that, in the absence of HP, ⍺1-microglobulin might take a predominant role in scavenging labile heme during malaria.

The median concentration of ⍺1-microglobulin in serum from children that developed severe non-cerebral *P. falciparum* malaria was significantly higher than that of children that developed uncomplicated *P. falciparum* malaria (Fig 5D). The median concentration of ⍺1-microglobulin in serum was indistinguishable in CM versus uncomplicated *P. falciparum*-infected children (Fig 5D). These observations suggest that ⍺1-microglobulin might “compensate” for the HP depletion and limit the accumulation of labile heme in serum in severe non-cerebral malaria.

Albumin concentration in serum from *Pcc*-infected mice was reduced by ∼50% (i.e., hypoalbuminemia), compared with noninfected controls (Fig 5E). This effect (i.e., hypoalbuminemia) was indistinguishable in *Pcc*-infected *Hp*^*−/−*^, *Hpx*^*−/−*^ and *Hp*^*−/−*^*Hpx*^*−/−*^ mice (Fig 5E). This suggests that the contribution of albumin to the heme buffering capacity of serum is reduced during *Plasmodium* infection.

The median concentration of albumin in serum was indistinguishable in uncomplicated *P. falciparum*-infected children versus CM versus children that developed severe non-cerebral *P. falciparum* malaria (Fig 5F).

The concentration of circulating low-density lipoprotein (LDL), a lipid/protein macromolecule that binds avidly to labile heme (Jeney et al, 2002), increased by ∼fivefold in adult *Pcc*-infected *Hp*^*+/+*^*Hpx*^*+/+*^, *Hp*^*−/−*^, *Hpx*^*−/−*^ and *Hp*^*−/−*^*Hpx*^*−/−*^ mice versus genotype and aged-matched noninfected controls (Fig 5G). The concentration of oxidized LDL was reduced by ∼30% (Fig 5H and I), accounting for a <10-fold lower ratio of oxidized versus total LDL in *Pcc*-infected versus noninfected genotype matched controls (Fig 5H and I). This suggests that *Plasmodium* infection is associated with major changes in the relative concentration and oxidation of plasma heme-binding proteins and macromolecules. To what extent this contributes to regulate the pathogenetic effects of labile heme during *P. falciparum* malaria remains, however, to be established.

### HP and HPX are essential to prevent malaria mortality in ageing mice

**Figure 6.**
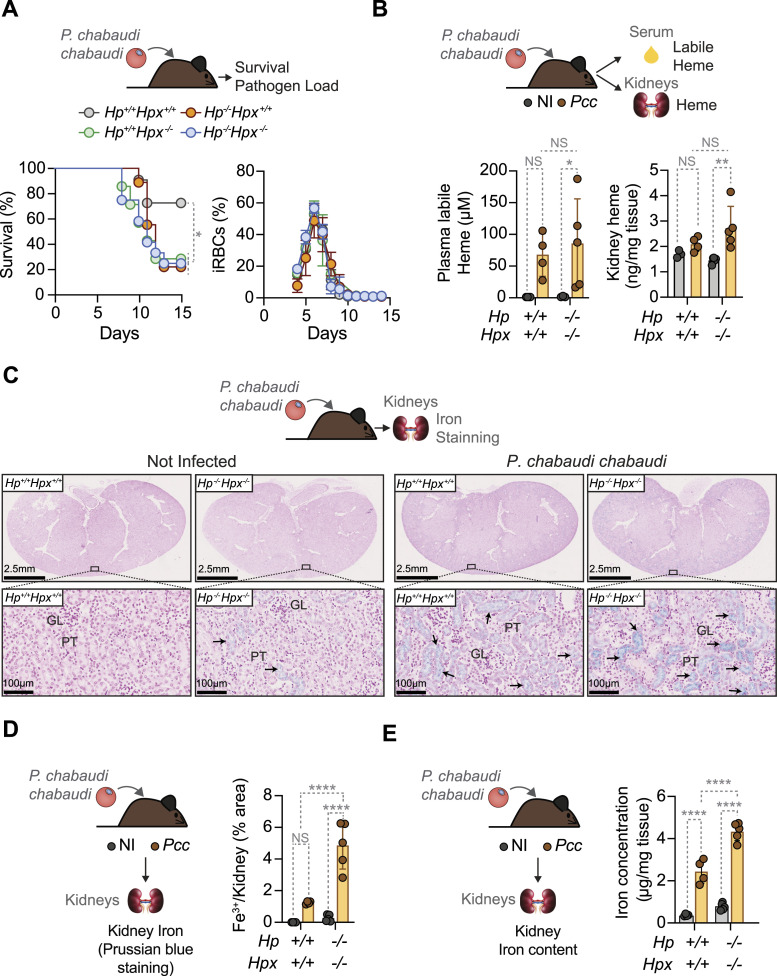
HP and HPX are essential to survive malaria in ageing mice. **(A)** Ageing (>30 wk) mice from the indicated genotypes were infected with *Pcc* (i.p., 2 × 10^6^ iRBCs). Survivals (left panel) are represented in the Kaplan–Meier plot and parasitemia (Right panel) by mean ± SD, monitored daily from day 3 post infection. Data pooled from three independent experiments (N = 7–12 mice *per* genotype), with a similar trend. **(B)** Quantification of labile heme in serum (Left panel) and renal heme (Right panel) at the peak of *Pcc* infection (2 × 10^6^ iRBC; day 7 post-infection) in *Hp*^*+/+*^*Hpx*^*+/+*^ and *Hp*^*−/−*^*Hpx*^*−/−*^ mice. Data shown as mean ± SD from one experiment (N = 4–5 mice *per* genotype). *P*-values determined using two-way ANOVA. **(C)** Kidney Perl’s Prussian blue staining (non-heme Fe^3+^) from N = 4–5 mice *per* genotype (non-infected or *Pcc*-infected: 2 × 10^6^ iRBC, at the peak of infection: day 7 post-infection) in one experiment. Top panels show whole-kidney sections and bottom panels higher magnifications from the area highlighted. Arrowheads indicate Fe^3+^ (blue). GL, glomerulus; PT, proximal tubules. **(C, D)** Quantification of renal non-heme Fe^3+^ accumulation, detected in the same experiment as in (C). Data presented as mean ± SD (N = 4–5 mice *per* genotype). **(E)** Quantification of renal non-heme iron concentration, shown as mean ± SD (N = 4–5 mice *per* genotype). *P*-values in (D, E) determined using two-way ANOVA. NS, nonsignificant; *P < 0.05; **P < 0.01; ****P < 0.0001. Source data are available for this figure.

In sharp contrast to adult mice (i.e., 8–12 wk), ageing (i.e., >30 wk) *Hp*^−/−^, *Hpx*^−/−^ and *Hp*^−/−^*Hpx*^−/−^ mice succumbed to *Pcc* infection, as compared with age-matched control *Pcc*-infected *Hp*^+/+^*Hpx*^+/+^ mice that survived (Fig 6A). This was not associated, however, with changes in parasite burden (Fig 6A), suggesting that HP and HPX are essential to establish disease tolerance to malaria (Medzhitov et al, 2012; Martins et al, 2019) in ageing, but not adult, mice.

### HP and HPX control renal iron overload and AKI in ageing mice

The concentrations of labile heme in serum and total heme in the kidneys from ageing *Pcc*-infected *Hp*^−/−^*Hpx*^−/−^ mice were in the range of control age-matched *Pcc*-infected *Hp*^+/+^*Hpx*^+/+^ mice (Fig 6B). However, ageing *Pcc*-infected *Hp*^−/−^*Hpx*^−/−^ mice accumulated higher levels of iron (Fig 6C–E) in the kidneys, including ferric (Fe^3+^) iron in renal proximal tubules, when comparing with *Pcc*-infected *Hp*^+/+^*Hpx*^+/+^ controls (Fig 6C and D). This suggests that HP and HPX limit the accumulation of heme–iron in the kidneys of ageing mice, a major driving force in the pathogenesis of malaria AKI (Ramos et al, 2019; Wu et al, 2023).

### Ageing alters renal response to *Plasmodium* infection

**Figure 7.**
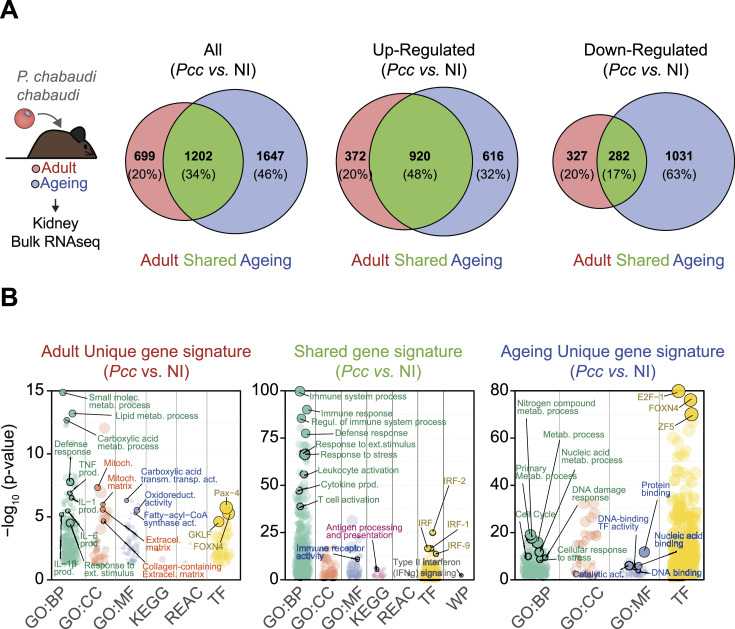
Age-dependent effect of HP and HPX on the regulation of gene expression in the kidneys of Pcc-infected mice. **(A)** Schematic representation of the experimental procedure used for renal bulk RNA-seq analyzes (Left panel). Euler plots of renal bulk RNA-seq data (Right panels) indicating the number and proportion of genes differentially expressed (All), induced (up-regulated) and repressed (down-regulated) in kidneys from *Pcc*-infected versus non infected (NI) male C57BL/6 mice. Red corresponds to genes differentially regulated, in a nonoverlapping manner (unique), in adult *Pcc*-infected male mice. Green corresponds to genes regulated, in an overlapping (shared) manner, in adult and ageing *Pcc*-infected mice. Blue corresponds to genes regulated, in a nonoverlapping manner (unique), in ageing *Pcc*-infected mice. **(A, B)** Manhattan plots of gProfiler renal bulk RNA-seq data from (A), depicting gene ontology (GO) analysis for biological processes (GO:BP; green), cellular components (GO:CC; orange), and molecular function (GO:MF; blue), Kyoto Encyclopedia of Genes and Genomes pathways (pink), Reactome (REAC, light green), transcription factors (TF; yellow), and WikiPathway (WP; gray). Left panel (red) corresponds to genes differentially regulated, in a nonoverlapping manner (unique), in adult *Pcc*-infected mice. Middle panel (green) corresponds to genes regulated, in an overlapping (shared) manner, in adult and ageing *Pcc*-infected mice. Right panel (blue) corresponds to genes regulated, in a nonoverlapping manner (unique), in ageing *Pcc*-infected mice. Data from N = 2 mice *per* experimental group from two independent experiments with similar trend. Source data are available for this figure.

To further understand the age-dependent protective effect of HP and HPX against malaria AKI, we compared bulk RNAseq data from adult versus ageing naïve and *Pcc*-infected *Hp*^+/+^*Hpx*^+/+^ mice. Consistent with previously described (Ramos et al, 2019; Wu et al, 2023), *Pcc* infection in adult *Hp*^+/+^*Hpx*^+/+^ mice was associated with a robust transcriptional response in the kidneys, as compared with age-matched noninfected controls (Fig 7A). This integrated (i.e., parenchyma plus hematopoietic cells) response was characterized by the induction of 1,292 genes and repression of 609 genes (Fig 7A). Ageing *Pcc*-infected *Hp*^+/+^*Hpx*^+/+^ mice also showed a robust transcriptional response (Fig 7A). However, only 34% of the 1,536 genes induced and 1,313 genes repressed in ageing *Pcc*-infected *Hp*^+/+^*Hpx*^+/+^ mice were shared with those induced or repressed in adult *Pcc*-infected *Hp*^+/+^*Hpx*^+/+^ mice, respectively (Fig 7A). This suggests that ageing interferes per se with the integrated renal transcriptional response to malaria.

The unique gene expression “signature” of adult *Pcc*-infected mice was related to proinflammatory cytokines (e.g., interleukin 1 and 6) and regulation of lipid metabolism as well as carboxylic metabolic processes (Fig 7B). These were associated with transcriptional programs regulated by the transcription factors Paired Box 4 (Pax4), Kruppel-like factor 4 (KLF4; gut-enriched Krüppel-like factor or GKLF) or Forkhead Box N4 (FOXN4) (Fig 7B).

Consistent with previously described (Wu et al, 2023), the shared gene-expression “signature” between *Pcc*-infected adult and ageing mice (Fig 7B) was related to type I and II interferon responses, and to antigen processing and presentation (Fig 7B). This was associated with transcriptional programs regulated by the interferon regulatory factor (IRF) family of transcription factors including IRF-1, 2 and 9 (Fig 7B).

Ageing *Pcc*-infected mice also presented a unique gene-expression “signature,” related to metabolism of nitrogen compounds and primary metabolic processes and with nucleic acid metabolic processes and stress responses, including DNA damage responses but also with cell cycle regulation (Fig 7B). This was associated with transcriptional programs regulated by the transcription factors E2F transcription factor 1 (E2F1), ZF5, and FOXN4 (Fig 7B).

These observations reveal that ageing interferes with the integrated renal transcriptional responses to *Plasmodium* infection without however, precipitating the onset of malarial AKI. Whether these transcriptional responses emanate predominatly from parenchyma or hematopoietic-derived cells is not clear.

### HP and HPX regulate the renal response to malaria in ageing mice

**Figure 8.**
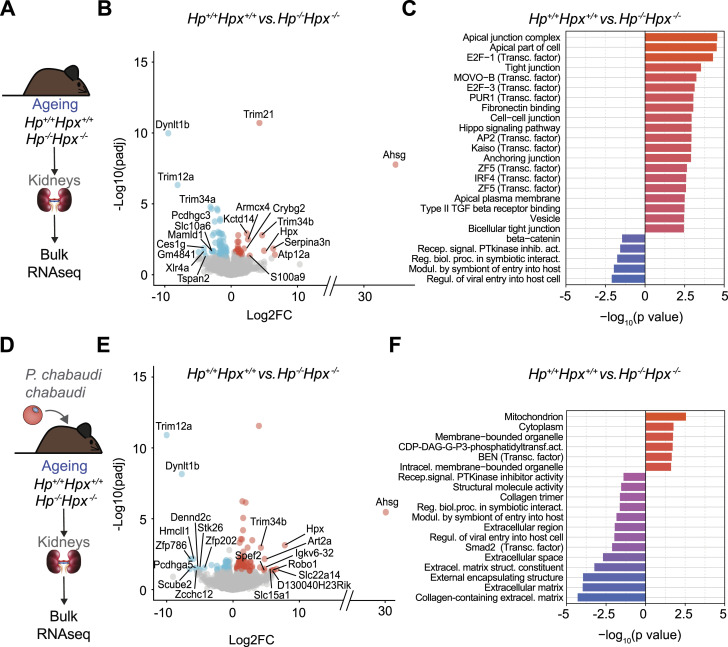
Age-dependent effect of HP and HPX on the regulation of gene expression in the kidneys of Pcc-infected mice. **(A)** Schematic representation of the experimental procedure used for renal bulk RNA-seq analyzes in ageing *Hp*^*−/−*^*Hpx*^*−/−*^ versus control aged-matched *Hp*^*+/+*^*Hpx*^*+/+*^ mice at steady state. **(B)** Volcano plots displaying differential gene expression between whole kidneys from ageing *Hp*^*−/−*^*Hpx*^*−/−*^ versus control aged-matched *Hp*^*+/+*^*Hpx*^*+/+*^ mice at steady state. **(B, C)** Gene ontology (GO) analysis of differentially expressed genes from (B). Top 20 significant up- or down-regulated GO terms depicted, for comparisons where more than 20 significant GO terms were found. **(D)** Schematic representation of the experimental procedure used for renal bulk RNA-seq analyzes in ageing *Pcc*-infected *Hp*^*−/−*^*Hpx*^*−/−*^ versus control aged-matched *Pcc*-infected *Hp*^*+/+*^*Hpx*^*+/+*^ mice. **(E)** Volcano plots displaying differential gene expression between whole kidneys from ageing *Pcc*-infected *Hp*^*−/−*^*Hpx*^*−/−*^ versus control aged-matched *Pcc*-infected *Hp*^*+/+*^*Hpx*^*+/+*^ mice. **(F)** Gene ontology (GO) analysis of differentially expressed genes from (E), displayed as in (C). Genes significantly up-regulated in kidneys from *Hp*^*−/−*^*Hpx*^*−/−*^ mice are represented in red dots, whereas blue dots represent down-regulated genes. Grey dots represent not statistically significant genes. Analysis of same data as Fig 7, performed using gProfiler. Data from N = 2–3 mice *per* experimental group from 1 independent experiment. Source data are available for this figure.

We asked whether HP and HPX shape the integrated renal transcriptional response to *Plasmodium* infection in ageing mice. Kidneys from ageing noninfected *Hp*^−/−^*Hpx*^−/−^ mice showed a distinct gene expression “signature,” driven by the induction of 29 genes and repression of 79 genes, compared with age-matched control *Hp*^+/+^*Hpx*^+/+^ mice (Fig 8A and B). Gene ontology analysis suggests that kidneys from ageing mice up-regulated the expression of genes associated with epithelial barrier integrity (e.g., apical junction complex; tight junction; cell-cell junction; anchoring junction, etc.), cell proliferation (e.g., *E2F-1*, *E2F-3*), apoptosis, and stem cell self-renewal (e.g., *Hippo signaling*) as well as with host–microbe interactions (e.g., regulation of symbiont or viral entry into host, etc.) (Fig 8C). This suggests that HP and HPX regulate steady state renal physiology.

The kidneys from *Pcc*-infected *Hp*^−/−^*Hpx*^−/−^ mice also showed a distinct gene-expression “signature” profile driven by the up-regulation of 98 genes and repression of 50 genes, compared with age-matched control *Pcc*-infected *Hp*^+/+^*Hpx*^+/+^ mice (Fig 8D and E). Gene ontology analysis suggests that among the up-regulated genes are mitochondrial genes, whereas the repressed genes were associated mainly with extracellular matrix (e.g., collagen trimer, extracellular region, extracellular space; extracellular matrix, structural constituent; external encapsulating structure, etc.) (Fig 8F). This suggests that ageing interferes with the renal transcriptional response to *Plasmodium* infection, associated with the onset of malarial AKI. To what extent the differential expression of these genes justifies how the combination of ageing and HP and HPX regulates the pathogenesis of malaria-associated AKI remains to be established functionally.

### Circulating HPX and heme are associated with *P. falciparum* AKI

**Figure 9.**
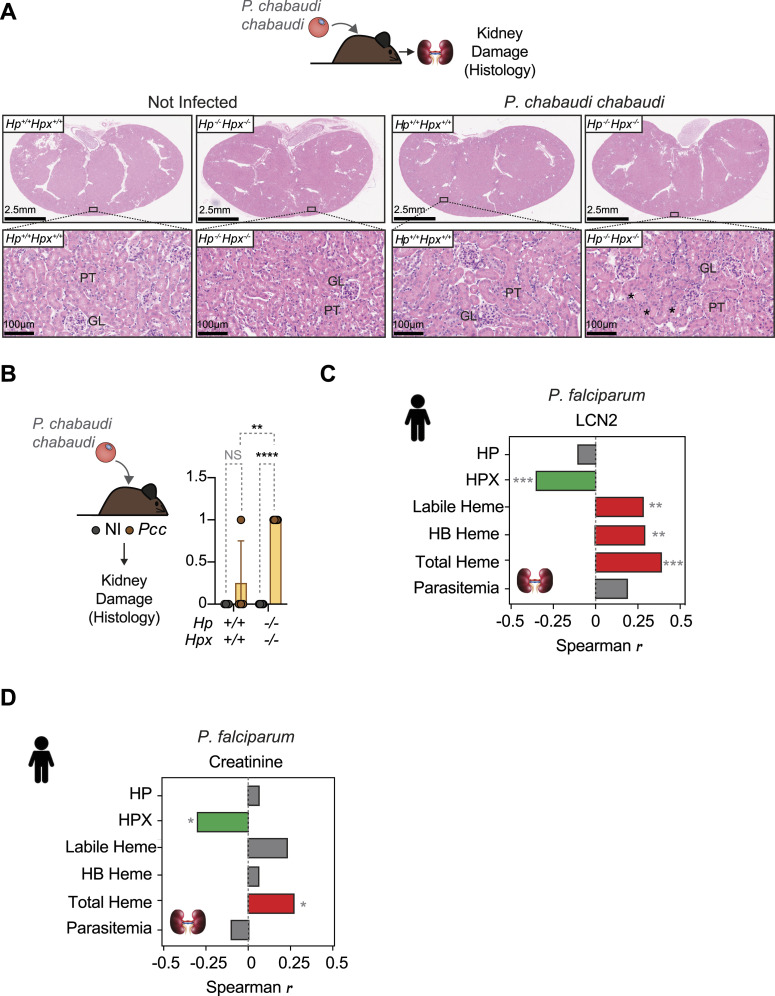
Levels of circulating HPX and heme are associated with *P. falciparum* acute kidney injury. **(A)** Representative H&E staining of the kidney from ageing (>30 wk) *Hp*^*+/+*^*Hpx*^*+/+*^ and *Hp*^*−/−*^*Hpx*^*−/−*^ mice infected with *Pcc* (i.p., 2 × 10^6^ iRBCs, at the peak of infection: day 7 postinfection). Images are representative from N = 4–5 mice *per* condition. Top panels show whole-kidney section and bottom panels show higher magnifications from the rectangle highlighted in the top panel. **(B)** Asterisks indicate hemoglobin casts. GL, glomerulus; PT, proximal tubules. **(A)** Histopathological evaluation of kidneys from (A) was performed using digitalized whole-slide images, corresponding to whole-kidney sections. Scores are represented as mean ± SD (n = 4–5 mice *per* genotype). Dots correspond to individual mice. *P*-values determined by Two-Way ANOVA. *P < 0.05; ****P < 0.0001; NS, not significant. Scores: 0 = No lesions; 1 = Single cell necrosis, discrete hemoglobin tubular casts; 2 = Mild; 3 = Moderate; 4 = Severe tubular cell necrosis, hemoglobin tubular casts. **(C, D)** Spearman correlation coefficients between indicated variables and LNC2 and creatinine in serum of *P. falciparum*-infected individuals (same individuals as in Fig 1), corrected for multiple tests (Holm–Sidak). Source data are available for this figure.

Ageing *Pcc*-infected *Hp*^−/−^*Hpx*^−/−^ presented more extensive HB cast nephropathy, predominantly in the proximal tubules, as compared with *Pcc*-infected *Hp*^+/+^*Hpx*^+/+^ mice (Fig 9A and B). This is consistent with renal iron overload promoting the pathogenesis of malaria AKI (Ramos et al, 2019; Wu et al, 2023).

We asked whether the levels of circulating HP, HPX, and/or labile heme were associated with *P. falciparum* AKI in the case-control study described in Table 1 (Sambo et al, 2010). HPX was negatively correlated with lipocalin 2 (LCN2) (*P* = 0.0001) (Fig 9C) and with creatinine (*P* = 0.006) (Fig 9D) concentrations in serum, two serological markers of AKI. These negative correlations remained significant when controlling for parasitemia (*P* = 0.000061 for LNC2 and *P* = 0.008 for creatinine) (Fig 9C and D).

Total heme, HB–heme, and labile heme were positively correlated with the concentration of LCN2 in the serum of *P. falciparum*-infected children (Fig 9C). These positive correlations remained significant when controlling for parasitemia (*P* = 0.000001 for total heme, *P* = 0.004 for HB-Heme and *P* = 0.0006 or labile heme) (Fig 9C). Although total heme was also positively correlated with creatinine concentration in serum (Fig 9D), this was no longer significant for labile heme or HB–heme (Fig 9D).

These observations suggest that HPX acts irrespectively of parasite burden to prevent labile heme from partaking in the pathogenesis of malarial AKI, contributing to the establishment of disease tolerance (Medzhitov et al, 2012; Martins et al, 2019) to *P. falciparum* malaria. The association of labile heme with CM (Fig 1D) and malaria AKI (Fig 9C and D) is consistent with the proposed pathophysiological contribution of AKI to the development of brain dysfunction in *P. falciparum* malaria (Conroy et al, 2023).

## Discussion

Having established that heme catabolism by HO-1 is protective against the pathogenesis of cerebral (Pamplona et al, 2007; Ferreira et al, 2008; Jeney et al, 2014) and non-cerebral (Seixas et al, 2009; Ramos et al, 2019) presentations of severe malaria in mice, we put forward that labile heme acts as a major driving force in the pathogenesis of severe presentations of malaria (Ferreira et al, 2008). However, two independent clinical studies have shown that microsatellite (GTn) polymorphisms in the human *HMOX1* promoter (i.e., lower GTn repeats), enhancing HO-1 expression, are associated with increased *P. falciparum* malaria susceptibility in children and adults (Takeda et al, 2005; Walther et al, 2012). This questioned whether the protective effects of HO-1 in rodent malaria are extrapolatable to the human disease. In support of the latter, there are “additional” *HMOX1* gene variants enhancing HO-1 expression that were associated with reduced susceptibility to *P. falciparum* CM in children (Sambo et al, 2010), consistent with experimental models of malaria in mice (Pamplona et al, 2007; Ferreira et al, 2008; Jeney et al, 2014).

The apparent discrepancy between the protective versus pathogenic effects of HO-1 in rodent models of malaria and human malaria, respectively, is likely explained by the opposing effects of HO-1, exerted at different stages of *Plasmodium* infection. Malaria transmission by *Anopheles* mosquitoes is associated with the induction of HO-1 by erythrophagocytic macrophages in the dermis, which limit the extent of damage imposed locally by microvascular bleeding (DeSouza-Vieira et al, 2020). Whether this impacts on the progression and outcome of malaria was, to the best of our knowledge, not established. As *Plasmodium* sporozoites migrate from the dermis to liver, HO-1 expression by Kupfer cells, and probably other cell compartments, becomes essential to establish the liver stage of malaria (Epiphanio et al, 2008). Thereafter, during the blood stage of infection, HO-1 acts in different cell compartments to prevent the developemnt of experimental CM (Pamplona et al, 2007; Ferreira et al, 2008; Jeney et al, 2014) and in renal proximal tubule epithelial cells (Ramos et al, 2022), to prevent the development of AKI, a major independent risk factor of malaria mortality in children and adults (Sitprija, 1988; Trang et al, 1992; Mishra & Das, 2008; Plewes et al, 2017; Cruz et al, 2018; Katsoulis et al, 2021; Wu et al, 2023). These findings suggest that heme catabolism by HO-1 exerts a dual role, promoting the initial stages of *Plasmodium spp.* infection whereas preventing, later on, the onset of severe presentations of malaria.

To probe the pathologic effect of labile heme in *P. falciparum* malaria we asked whether targeting extracellular HB and/or labile heme by HP and HPX, respectively, limit the accumulation of labile heme and/or prevent the pathogenesis of severe presentations of malaria. Our findings suggest that labile heme is a major risk factor for severe presentations of *P. falciparum* malaria in children (Table 1, Fig 1). This is not the case however, for HP nor HPX (Table 1, Fig 1C), suggesting that other heme-binding proteins and macromolecules might contribute to control the pathogenetic effects of circulating labile heme. In strong support of this notion, HP and HPX have little or no contribution to the heme-buffering capacity of mouse serum (Fig 5A and B). As the assay used to quantify heme-buffering capacity is based on a heme-binding sdAb with an affinity towards heme in the range of 10^−7^ M (Gouveia et al, 2017), our findings suggest that a number of plasma heme-binding proteins and/or macromolecules bind labile heme with an affinity higher than 10^−7^ M. These are likely to include circulating α_1_-microglobulin (De Simone et al, 2023), which accumulates to a higher extent in plasma from children developing non-cerebral severe presentations, when compared with uncomplicated *P. falciparum* malaria (Fig 5D). This is not observed for albumin (Fig 5F), which binds labile heme with an affinity in the range of 10^−8^ M (Adams & Berman, 1980; Severance & Hamza, 2009). However, this should not exclude albumin from acting as an intermediate low-affinity high-capacity heme scavenger (Bunn & Jandl, 1968; Ascenzi et al, 2005; De Simone et al, 2023) during *P. falciparum* malaria.

Other putative protective heme scavengers controlling the pathogenic effects of labile might include LDL, which binds labile heme (Jeney et al, 2002) with an affinity in the range of 10^−11^–10^−12^ M (Camejo et al, 1998). The marked increase in the concentration of LDL in serum of *Pcc*-infected mice (Fig 5G), suggests that LDL might provide an alternative heme-scavenging route during malaria. In support of this notion, labile heme binds and induces lipid peroxidation in LDL (Jeney et al, 2002), causing nonenzymatic cleavage of the protoporphyrin ring of heme and retaining iron (Balla et al, 1991). To what extent LDL contributes individually or collectively with albumin and/or α_1_-microglobuling to mitigate the pathogenic effects of labile heme during malaria remains however to be established.

We note that despite the accumulation of labile heme in plasma (Fig 2E and F), this was not associated with the accumulation of oxidized LDL (Fig 5H and I), at the peak of *Pcc* infection. This suggests that heme-driven LDL oxidation (Jeney et al, 2002) is actively prevented during *Plasmodium* infection, via a mechanism that remains to be established.

The age-dependent protective effect of HP and/or HPX against malaria AKI in mice (Figs 6–9) and the inverse correlation of HPX and heme with renal impairment in *P. falciparum* malaria (Fig 9C and D) are consistent with the age-dependent increase in susceptibility to renal impairment in *P. falciparum* malaria (Dondorp et al, 2008). Moreover, the age-dependent renal transcriptional response (Fig 7) and protective effect of HP and HPX (Fig 8) in *Plasmodium*-infected mice supports the idea of an age-dependent impairment of tissue damage control mechanisms (Soares et al, 2014) establishing disease tolerance to malaria (Martins et al, 2019; Ramos et al, 2019). This interpretation is contingent on the accuracy of the quantitative approach (i.e., microscopy) used to estimate peripheral parasitemia in clinical samples and on peripheral parasitemia reflecting parasite biomass. Of note, the notion of an age-dependent impairment of disease tolerance to malaria is in keeping with the age-dependent impairment of disease tolerance to bacterial sepsis (Sanchez et al, 2023 *Preprint*).

An open question raised by our study pertains to the possible pharmacologic use of HP and HPX in the treatment of severe *P. falciparum* malaria, presumably, as an adjunctive therapy with standard antimalarial drugs. Although this remains to be tested, such an approach warrants some considerations. Namely, sustained oxidation of extracellular HB can generate meth-(Fe^3+^)HB and ferryl-(Fe^4+^)HB. The latter forms covalently linked multimeric complexes (Vallelian et al, 2008) that can “escape” HP and activate endothelial cells (Silva et al, 2009; Nyakundi et al, 2019; Erdei et al, 2020), presumably therefore contributing to the pathogenesis of severe presentations of malaria.

Moreover, damaged RBC can release heme-containing microvesicles, described originally in sickle cell anemia (Camus et al, 2012) and thereafter in malaria (Pais et al, 2022). This suggests that a fraction of the heme released from damaged RBC might “escape” HPX, presumably therefore contributing to the pathogenesis of severe presentations of malaria, as demonstrated for experimental CM in mice (Pais et al, 2022).

More recently, heme-binding histidine-rich protein II (HRPII) nanomers, secreted by *P. falciparum*-infected RBC, were shown to induce vascular inflammation and edema (Nguyen et al, 2023). To what extent these partake in the pathogenesis of severe presentations of malaria is likely but remains to be tested experimentally.

In conclusion, circulating labile heme is a risk factor for severe presentations of *P. falciparum* malaria, consistent with the functional role of labile heme in the pathogenesis of severe malaria in mice (Pamplona et al, 2007; Ferreira et al, 2008; Gozzelino et al, 2010; Ramos et al, 2019; Wu et al, 2023). Whereas HP and HPX exert some level of control over the pathogenic effects of labile heme, other serum heme-binding proteins and/or macromolecules might partake in this defense mechanism that establishes disease tolerance to malaria. Identifying and characterizing such heme-binding proteins and/or macromolecules should contribute to the development of much needed therapeutic approaches against CM and non-cerebral malaria severe presentations of *P. falciparum* malaria.

## Materials and Methods

### Human *P. falciparum* malaria data and analyses

#### Subjects

A subset of 123 children, from a previously described case-control study (Sambo et al, 2010) comprising a total of 749 children ranging from 6 mo to 13 yr of age, were analyzed in the present study. These included 58 children presenting cerebral malaria (CM), 61 presenting non-cerebral malaria severe malaria and 25 developing non-severe (uncomplicated) malaria. Patients were selected among attendance to the Hospital Pediátrico David Bernardino, Luanda, Angola and ethical permission was granted by an ethical committee appointed by the Angolan Ministry of Health. Written, informed consent was obtained from the parents or child guardians. Sample collection was carried out from February 2005 to May 2007.

#### Phenotypic and clinical criteria

Malaria was diagnosed based on a positive asexual parasitemia detected on a Giemsa-stained thick smear. For parasitemia quantification, the number of parasites per 100 high-power microscopic fields was estimated and the parasite density was calculated (Greenwood & Armstrong, 1991). CM was defined according to the WHO criteria: a coma score <3 in Blantyre Scale for children <60 mo or a coma score <7 in Glasgow Scale for children ≥60 mo. Meningitis and encephalitis were ruled out by cerebrospinal fluid analysis after lumbar puncture. Exclusion criteria included a different known etiology of encephalopathy and hypoglycemia (glycaemia < 40 mg/dl). A fraction of CM patients showed additional clinical complications such as severe malaria anemia and hyperparasitemia. Children with non-cerebral malaria severe malaria included patients with severe malaria anemia (HB <5 g/dl or hematocrit <15%) and/or hyperparasitemia (≥100 parasitized RBC per high-power microscopic field). Patients with consciousness disturbances or with other disease were excluded. The uncomplicated malaria group represents outpatients with malaria diagnosis and febrile illness without any clinical finding suggestive of other causes of infection and with no manifestations of severe malaria. Patient treatments followed the established hospital guidelines.

#### Genotyping

Genomic DNA was extracted from whole blood using the Chemagen Magnetic Bead Technology. DNA was quantified in the individual sample preparations using PicoGreen reagents according to the supplier instructions. 11 SNPs were identified in public databases (dbSNP 126, Ensemble release 45) by selecting for minor allele frequency higher than 5% in HapMap Yoruba population (sub-Saharan African). The SNP genotyping method used the Mass Array software to design multiplex reactions for PCR and iPlex primer extension (Sequenom) and the MALDI-TOF-based Mass Array genotype platform (Sequenom). Genotyping quality control excluded from further analysis one SNP that was monomorphic (rs17880288). 10 SNPs that passed quality control criteria were spanning the *HMOX1* gene including the 5′ region, the structural gene and the 3′ region.

#### Quantification of cell-free HB, heme, HP, HPX, albumin, and α1-mG in human samples

Concentration of cell-free HB in serum was determined by spectroscopy. Briefly, visible spectra of undiluted serum samples were taken with a BioTek Synergy H1 plate reader. Optical density was measured at λ_577_ and λ_630_ nm with background subtraction, and the concentration of HB-bound heme in serum was calculated (μmol/liter) as follows: HB-heme = 10x(66xODλ_577_ − 80xOD λ_630_). Total serum heme was measured using the 3,3′,5,5′ tetramethylbenzidine (TMB) peroxidase assay (BD Biosciences). Briefly, serum sample (1 μl) was mixed TMB reagent (200 μl) and incubated 30 min in dark. Absorbance was measured at λ_655_. Purified HB was used as standard, to calculate heme concentration in serum. Concentration of labile heme in serum was calculated as follows: Labile heme = (Total heme) − (HB-heme). The concentration of HP and HPX in serum was measured by ELISA (Alpco), as described (Larsen et al, 2010). The concentration of albumin (Novus Biologicals) and α1-microglobulin (Abcam) in serum were measured by ELISA (Alpco), as *per* the manufacturer’s instructions.

#### Logit regressions

For each pairwise comparison of each of the subgroups (CM, Severe non-Cerebral, and Uncomplicated Malaria), we set up a logit regression of each of the infection parameters (Parasitemia, HP, HPX, Total Heme, Labile Heme, Hb Heme) after standardization (enforcing zero mean and unit SD), with Age and Gender as co-variates (Fig 1D). To establish heme as independent risk factor, a logit regression was set up that in addition to age and gender, included the other relevant variables.

#### HP and HPX correlations with heme

Ordinary least squares regression was used to assess correlation of Labile Heme with HP and HPX, controlling for Age, Gender, and Parasitemia.

#### Kidney damage correlations

Correlation coefficients between HP, HPX, total heme, HB-heme, labile heme, parasitemia, and LNC2 or creatinine were calculated through Spearman rank correlations. *P*-values were corrected for multiple tests using Holm–Sidak method.

#### Mice and gene deletions

Mice were bred and maintained under specific pathogen-free conditions at the Instituto Gulbenkian de Ciência, Oeiras. Protocols were approved by the Ethics Committee of the Instituto Gulbenkian de Ciência (A008.2010 and A009.2011) and by the Portuguese National Entity (008959 and 018071; Direcção Geral de Alimentação e Veterinária). All experiments were performed according to the Portuguese (Decreto-Lei 113/2013) and European (Directive 2010/63/EU) legislations. Hp deficient (*Hp*^*−/−*^) and Hpx-deficient (*Hpx*^*−/−*^) C57BL/6 mice (Tolosano et al, 2002) were provided originally by Dr. Emanuela Tolosano (Molecular Biotechnology Center, University of Torino) and were bred at the Instituto Gulbenkian de Ciência. HP deletion has a small but significant adverse effect on the postnatal viability (Lim et al, 1998). *Hp*^*−/−*^ and *Hpx*^*−/−*^ C57BL/6 mice were intercrossed to generate *Hp*^*−/−*^*Hpx*^*−/−*^ C57BL/6 mice. BALB/c *Hmox1*^*−/−*^ mice were originally obtained from Shaw-Fang Yet (Yet et al, 1999), backcrossed in heterozygosity into the C57BL/6 background and crossed with *Hp*^*−/−*^*Hpx*^*−/−*^ mice to generate *Hp*^*−/−*^*Hpx*^*−/−*^*Hmox1*^*+/−*^ mice.

#### Parasites, infection and parasitemia

Mice were infected (i.p.; 10^5^ or 2 × 10^6^ infected RBC) as described (Pamplona et al, 2007; Seixas et al, 2009). Transgenic GFP-expressing *P. berghei* ANKA (Pamplona et al, 2007), *P. berghei* NK65 (Pamplona et al, 2007), *P. yoelii yoelii* 17XNL or *P. chabaudi chabaudi* AS have been previously described (Gouveia et al, 2017; Ramos et al, 2019). The day of infection was considered day zero (D0). Parasitemia (i.e., percentage of infected RBC; iRBC) was assessed by microscopic visualization of Giemsa-stained blood smears in a total of four fields (1,000× magnification) as described (Seixas et al, 2009). For *Pb* ANKA infections, parasitemia was determined by flowcytometry (FACScan; Becton Dickinson) and analyzed using the FlowJo software (Tree Star) (Pamplona et al, 2007). Of note, *Pcc* infection severity and kinetics change with inoculum, resulting in a peak of parasitemia at 8–9 d post infection when mice are infected initially with 1 × 10^5^ iRBC and at 6–7 d post infection when infected with 2 × 10^6^ iRBC.

#### Heme quantification in mouse samples

Total heme was quantified in serum using the formic acid assay. Briefly, serum (1 μl) was diluted in H_2_O (50 μl) to which formic acid was added (150 μl). Hemin serial dilutions were used as standards. Absorbance was determined at λ_405_ and λ_490_ as background subtraction. Bioavailable heme was quantified in mouse serum samples as described (Gouveia et al, 2017; Ramos et al, 2019). Total heme in organs was quantified using the oxalic acid method (Marcero et al, 2016). Briefly, ∼10 mg organ pieces were weighed and homogenized in 120 µl PBS using metal beads and a QIAGEN TissueLyser II. Tissue homogenates (20 µl) were placed in a flat bottom 96-well plate, and 200 µl of warm, concentrated oxalic acid (1.5 M) was added to each sample. Standard heme concentrations (3.125–800 ng/ml) were prepared and diluted in oxalic acid (1.5 M). Samples and standard concentrations were acid-hydrolyzed for 30 min at 95°C and briefly spun down. Sample and standard curve supernatants were transferred (200 µl) to a fresh flat bottom 96-well plate and fluorescence was assessed (ex. λ_400_ nm, em. λ_662_ nm, chamber at 37°C) using a BioTek Synergy H1 plate reader. Background signal was subtracted, and sample concentrations were calculated based on the standard concentration curve and normalized to tissue weight.

#### Spectrum analysis of human plasma samples

Human plasma samples from patients infected with *P. falciparum* were diluted 2x in PBS. and placed in a flat-bottom 96 well plate. Absorbance spectrum was measured between 350–750 nm and PBS was recorded as blank measurement, using a BioTek Synergy H1 plate reader.

#### Iron (non-heme) quantification in mouse samples

Total non-heme iron was quantified in serum as before (Wu et al, 2023). Briefly, ∼20 mg tissue pieces were weighed and homogenized in 120 µl PBS using metal beads and a QIAGEN TissueLyser II. Sample homogenates (100 µl) were mixed with 50 µl of 300 mg/ml trichloroacetic acid in 29.5% hydrochloric acid and heated overnight to 65°C for acid hydrolysis. Samples were centrifuged (3,000*g*, 5 min) to clarify the lysate and the supernatant (60 µl) was transferred to a fresh 96-well flat bottom plate. Standard concentrations of iron were prepared by dissolving iron (II) sulfate heptahydrate in distilled water and by sequential dilutions of the stock concentration (range 22.4–0.0875 μg/ml) and placed in duplicates in the same plate as the samples. Colorimetric reagent was prepared fresh by mixing a solution of 347 mg/ml of sodium acetate with a solution of 3.4 mg/ml bathophenanthroline disulfonic acid and 4.4 mg/ml ascorbic acid, at a ratio of nine to one, respectively (final concentration of 323, 3 mg/ml sodium acetate; 0.34 μg/ml bathophenanthroline disulfonic acid; and 0.44 mg/ml ascorbic acid). Samples and standard concentrations were mixed with 160 µl colorimetric reagent, and absorbance at 539 nm was immediately assessed using a BioTek synergy H1 plate reader. Background signal was subtracted, and concentrations were calculated using the standard curve and normalized to tissue weight.

#### Serology

Mice were euthanized at the indicated time points after infection and blood was collected for serological analysis. Serum was obtained from two consecutive centrifugations (1,600*g*, 5 min, 4°C). Creatinine, urea, aspartate aminotransferase, and alanine aminotransferase concentrations in serum were determined by DNAtech (https://dnatech.pt/). HP (Life Diagnostics), HPX (Life Diagnostics), LDL, oxLDL, and α-1 microglobulin (Wuhan USCN Business Co., Ltd), and albumin (Bethyl Labs) concentrations in serum were measured by ELISA, according to the manufacturer’s instructions.

#### Histopathology

Mice were euthanized at the indicated time points after *Pcc* infection and perfused in toto with ice-cold PBS. Organs were harvested, fixed in 10% formalin, embedded in paraffin, sectioned, and stained with hematoxylin and eosin or using Perl’s staining, to detect Fe^3+^ in tissues. Whole sections were analyzed in a DMLB2 microscope (Leica); images were acquired with a DFC320 camera (Leica) and a NanoZoomer-SQ Digital slide scanner (Hamamatsu Photonics). Images were reconstructed using the NDP.view2 (Hamamatsu Photonics) software. Histopathology analyzes were performed by Dr. Pedro Faísca (IGC Histopathology Unit). For iron (Fe^3+^) quantification in kidney sections, the fraction of iron positive staining in the total area of the kidney was quantified using the color threshold plugin of the ImageJ software (Rasband, W.S., ImageJ, U.S. NIH, Bethesda, Maryland, USA).

#### HP and HPX expression

C57BL/6J mice were euthanized at steady state or 7 d after *Pcc* infection and organs were harvested. *Hp* and *Hpx* mRNA expression were determined by qRT–PCR, as described above. Briefly, organs were harvested and snapped frozen in liquid nitrogen. Total RNA was isolated from mouse organs using tripleXtractor reagent (#GB23.0100; GRiSP). RNA (1 µg) was used to synthesize cDNA (Xpert cDNA Synthesis Mastermix; #GK81.0100; GRiSP) and qRT-PCR was performed with iTaq Universal SYBR Green Supermix (Bio-Rad). Transcript number was calculated from the threshold cycle (Ct) of each gene with a 2^−ΔCT^ method (relative number) and normalized to acidic ribosomal phosphoprotein P0 (*Arbp0*). Primers: *Arbp0* Fwd: 5′-CTTTGGGCATCACCACGAA-3′ and *Arbp0* Rev: 5′-GCTGGCTCCCACCTTGTCT-3′; *Hp* Fwd: 5′-AAACTCCCCGAATGTGAGGC-3′ and *Hp* Rev: 5′-TCCATAGAGCCACCGATGATG-3′, *Hpx* Fwd: 5′-GTACCCGAACACTGCTTGGA-3′ and *Hpx* Rev: 5′-CCTCGCTGAGATCAACTCCC-3′. Alternatively, *Hp* and *Hpx* expression was assessed in livers of fasted (non-infected) or *Pcc*-infected C57BL/6J mice, by bulk RNA sequencing analyses. Data for normalized gene expression were retrieved from a previously published dataset (Ramos et al, 2022).

#### Bioavailable heme

The assay was performed essentially as previously described (Gouveia et al, 2017). Briefly, bioavailable heme was measured using a previously described cellular heme reporter assay based on heme-dependent HRP activity (White et al, 2013; Yuan et al, 2016). Briefly, HEK293 cells (ATCC; 5 × 10^4^ cells/well in a 24 well plate) were grown overnight (DMEM, 10% FBS, 1% penicillin 10,000 U/ml, streptomycin 10,000 μg/ml) and transiently transfected (4–6 h, Lipofectamin 2000; Invitrogen) with an expression vector encoding the HRP gene under the control of the EF-1α or a control vector in opti-MEM reduced serum (Gibco by Thermo Fisher Scientific), as described (White et al, 2013; Yuan et al, 2016). Peroxidase activity was quantified using 3,3′,5′-Tetramethylbenzidine (TMB) substrate reagent set (BD OptEIA by Thermo Fisher Scientific) and normalized to protein expression (Quick Start Bradford Protein Assay; Bio-Rad). HRP concentration was determined based on Beer–Lambert law using the extinction coefficient (λ_403nm_; E_mM_ = 100).

#### Serum heme buffering capacity

The assay was performed essentially as previously described (Gouveia et al, 2017). Briefly, increasing concentrations of hemin were incubated with mouse serum (1:30H, RT; agitation). Labile heme (not bound to serum protein or macromolecules with an affinity >10^−7^ M) was detected with the heme-specific SdAb 1A6 (Gouveia et al, 2017). The SdAb 1A6 (0.3–5 μg/ml) was bound to 96-well plate via incubation in 50 mM carbonate/bicarbonate buffer (pH 9.6, 16 h, 4°C). The plate was washed (5x, PBS 0.1% Tween 20) and blocked (2 h, RT) with protein free blocking buffer. Hemin (0.15–5 μM in PBS) was used as standard. Plates were washed (5x, PBS, 0.1% Tween 20) and heme was detected using biotinylated heme-specific 2H7 sdAb (2.5–5 ng/μl) in PBS. Plates were washed (5x, PBS, 0.1% Tween 20) and biotinylated sdAb was detected using Alkaline phosphatase conjugated with ExtrAvidin (1/2,500; Sigma-Aldrich) in protein blocking buffer. Plates were washed (5x, PBS 0.1% Tween 20) and alkaline phosphatase was revealed using pNPP (1 mg/ml; Sigma-Aldrich). Absorbance was measured in a microplate reader at λ_450nm_. The concentration of heme required to reduce by 50% the serum heme-binding capacity is defined as heme-buffering capacity (HBC)_1/2_.

#### Bulk RNA sequencing and analysis of kidney samples

Kidneys were harvested from euthanized *Hp*^*+/+*^*Hpx*^*+/+*^ or *Hp*^*−/−*^*Hpx*^*−/−*^ mice, 7 d after *Pcc* AS infection and organs were harvested and snapped frozen in liquid nitrogen. Total RNA was isolated from mouse organs using tripleXtractor reagent (#GB23.0100; GRiSP), chloroform, isopropanol, and ethanol, according to manufacturer’s instructions. Full-length cDNAs and sequencing libraries were generated following the SMART-Seq2 protocol, as previously described (Picelli et al, 2014). The quality control was performed (Agilent Technologies), and after the samples passed the quality check the library preparation including cDNA “tagmentation,” PCR-mediated adaptor addition and amplification of the adapted libraries was performed following the Nextera library preparation protocol (llumina Tagment DNA Enzyme and Buffer, Illumina #20034211; KAPA HiFi HotStart ReadyMix, Roche #07958935001; Nextera XT Index Kit v2 Set A, Illumina #15052163; Nextera XT Index Kit v2 Set D, Illumina #15052166), essentially as previously described (Baym et al, 2015). Libraries were sequenced (NextSeq2000 sequencing; Illumina) using 100 SE P2 and sequence information was extracted in FastQ format, using Illumina DRAGEN FASTQ Generation v3.8.4, producing around 25–65 million reads per sample. Library preparation and sequencing were performed at the Instituto Gulbenkian de Ciência Genomics Unit. The fastq reads were aligned against the mouse reference genome GRCm39 using the annotation GENCODE M28 (STAR; v.2.7.9a) (Dobin et al, 2013). FeatureCounts (REF) (v.2.0.0) (Liao et al, 2014) was used to perform read summarization by assigning uniquely mapped reads to genomic features. Gene expression tables were imported into the R programming language and environment (v.3.6.3) to perform differential gene expression and data visualization. Differential gene expression was performed using the DESeq2 R package (Love et al, 2014) (v.1.31.7). Gene expression was modeled by the following linear design ∼ genotype + treatment + age + genotype:treatment + genotype:age + treatment:age + genotype:treatment:age including the following three factors for the samples: treatment (two levels: Non infected n = 9 and *Pcc* infected n = 9), age (two levels: Ageing n = 10 and Adult n = 8) and genotype (two levels: *HpHpx* mutant n = 8 and WT n = 10). Genes not expressed or presenting an average expression inferior to five counts across the 18 samples were removed resulting in 14,073 genes for downstream differential gene expression analysis. We subsequently ran the function DESeq which estimates the size factors by estimateSizeFactors, dispersion (by estimateDispersions) and fits a binomial GLM fitting for βi coefficient and Wald statistics (by nbinomWaldTest). Finally, the pairwise comparisons tested through contrasts with the function results, given the alpha of 0.05, were: Non infected *Hp*^*−/−*^*Hpx*^*−/−*^ versus control aged-matched; non-infected *Hp*^*+/+*^*Hpx*^*+/+*^ ageing mice; *Pcc*-infected *Hp*^*−/−*^*Hpx*^*−/−*^ versus control aged-matched; *Pcc*-infected *Hp*^*+/+*^*Hpx*^*+/+*^ ageing mice; *Pcc*-infected *Hp*^*+/+*^*Hpx*^*+/+*^ versus control aged-matched non-infected *Hp*^*+/+*^*Hpx*^*+/+*^ ageing mice; *Pcc*-infected *Hp*^*+/+*^*Hpx*^*+/+*^ versus control aged-matched non infected *Hp*^*+/+*^*Hpx*^*+/+*^ adult mice. Differentially expressed genes are genes with an adjusted *P*-value < 0.05 and an absolute log_2_ fold change > 0.58 (1.5-fold). Normalized gene expression counts were obtained with the function counts using the option normalized = TRUE. Volcano plots were done with the ggplot2 R package (v.3.3.2) (Wickham, 2016).

Euler plots representing the overlapping differentially regulated genes between pair-wise comparisons were generated using the “eulerr” R package (v7.0.0) (Larsson et al, 2022). Functional enrichment analysis was performed with the gprofiler2 R package (v.0.2.2) (Kolberg et al, 2020). Enrichment was performed using the function *gost* based on the list of up- or down-regulated genes genes with an adjusted *P*-value < 0.05 and an absolute log_2_ fold change > 0.58 (1.5-fold), between each pairwise comparison (independently), against annotated genes (domain_scope = “annotated”) of the organism *Mus musculus* (organism = “mmusculus”). Gene lists were sorted according to adjusted *P*-value (ordered_query = TRUE) to generate Gene Set Enrichment Analysis style *P*-values. Only statistically significant (user_threshold = 0.05) enriched functions are returned (significant = TRUE) after multiple testing corrections with the default method g:SCS (correction_method = “analytical”). The gprofiler2 queries were run against all the default functional databases for mouse which include: Gene Ontology (GO:MF, GO:BP, GO:CC), Kyoto Encyclopedia of Genes and Genomes, Reactome (REAC), TRANSFAC (TF), miRTarBase (MIRNA), Human phenotype ontology (HP), WikiPathways (WP), and CORUM (CORUM). For future reference, gprofiler2 was performed using database versions Ensembl 110, Ensembl gene 57 (database updated on 09/20/2023).

## Data Availability

Bulk RNAseq data from this publication were deposited on the Gene Expression Omnibus database (GEO, Ncbi; https://www.ncbi.nlm.nih.gov/geo/), with the assigned identifier GSE253390.

## Supplementary Material

Reviewer comments
